# Microstructured gas-liquid-(solid) interfaces: A platform for sustainable synthesis of commodity chemicals

**DOI:** 10.1126/sciadv.ado5448

**Published:** 2024-05-29

**Authors:** Kang Wang, Marc Pera-Titus

**Affiliations:** Cardiff Catalysis Institute, Cardiff University, Cardiff CF10 3AT, UK.

## Abstract

Gas-liquid-solid catalytic reactions are widespread in nature and man-made technologies. Recently, the exceptional reactivity observed on (electro)sprayed microdroplets, in comparison to bulk gas-liquid systems, has attracted the attention of researchers. In this perspective, we compile possible strategies to engineer catalytically active gas-liquid-(solid) interfaces based on membrane contactors, microdroplets, micromarbles, microbubbles, and microfoams to produce commodity chemicals such as hydrogen peroxide, ammonia, and formic acid. In particular, particle-stabilized microfoams, with superior upscaling capacity, emerge as a promising and versatile platform to conceive high-performing (catalytic) gas-liquid-(solid) nanoreactors. Gas-liquid-(solid) nanoreactors could circumvent current limitations of state-of-the-art multiphase reactors (e.g., stirred tanks, trickle beds, and bubble columns) suffering from poor gas solubility and mass transfer resistances and access gas-liquid-(solid) reactors with lower cost and carbon footprint.

## Teaser

Microstructured gas-liquid-(solid) interfaces substantially accelerate chemical reactions in comparison to bulk phases.

## INTRODUCTION

**Fig. 1.**
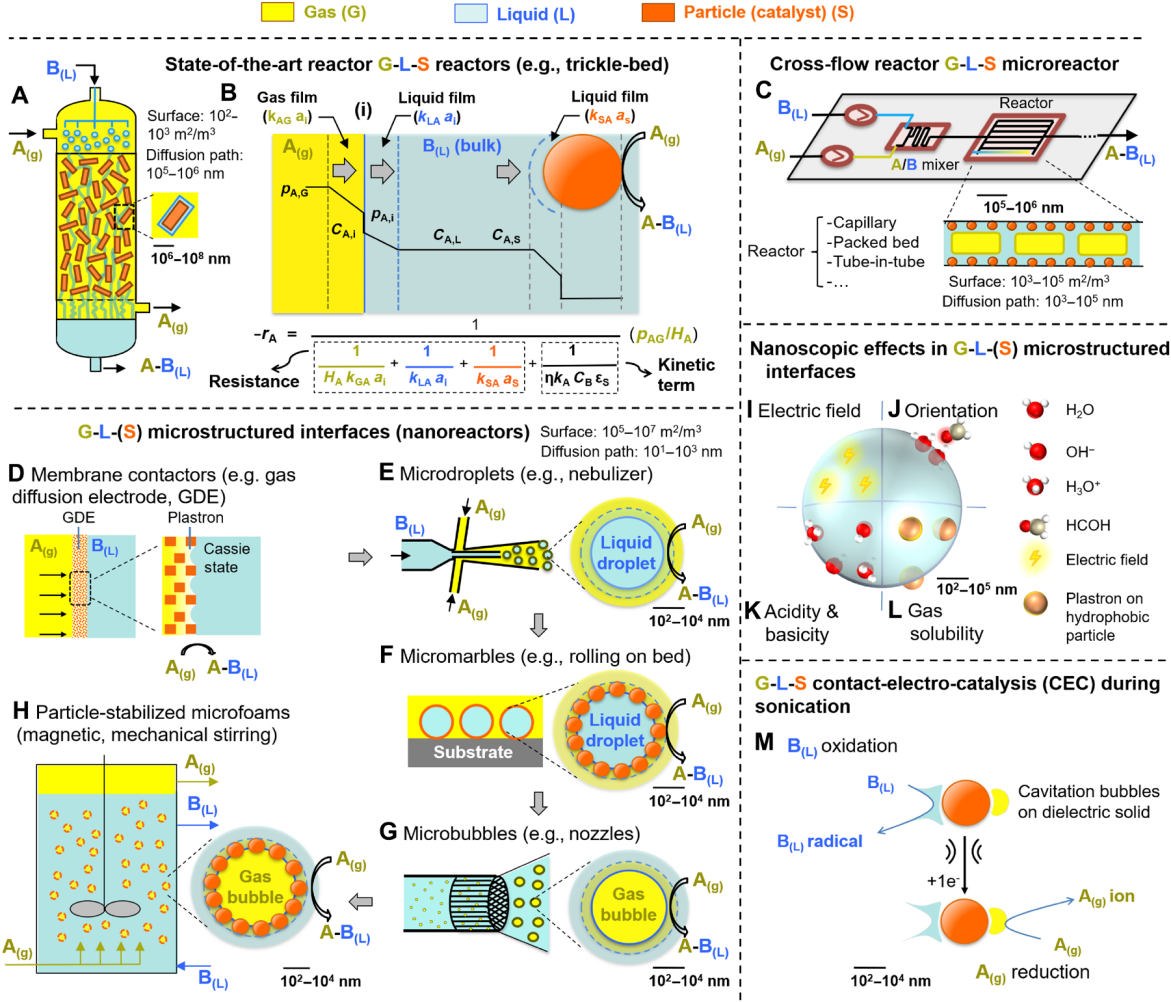
State-of-the-art G-L-(S) reactors. (**A**) Trickle-bed reactor. (**B**) Representative gas transfer profile and reactor rate in G-L-S reactors assuming an excess of liquid reactant B and a second-order reaction with an intrinsic reaction rate −*r*_A_ = *k*_A_*C*_A_*C*_B_ (*C*, concentration; *p*_A_, gas pressure; *H*_A_, Henry’s solubility constant; *k*_A_, mass transfer coefficient of the gas; *k*, kinetic constant; η, catalyst effectiveness; ε_S_, % solid loading). (**C**) Cross-flow microreactor. Microstructured G-L-(S) interfaces (nanoreactors): (**D**) membrane contactors, (**E**) microdroplets, (**F**) micromarbles, (**G**) microbubbles, and (**H**) particle-stabilized microfoams. Nanoscopic effects in microstructured G-L-(S) interfaces: (**I**) enhanced electric fields, (**J**) preferential orientation of reagents and water, (**K**) increased acidity or basicity, and (**L**) enhanced gas concentration using plastrons. (**M**) Contact-electro-catalysis (CEC) between gas-liquid and a (fluorinated) dielectric solid.

Gas-liquid-solid (G-L-S) catalytic reactions are widespread in the chemical and pharmaceutical industries and in environmental chemistry (*1*, *2*). The reactions are conditioned by the very low gas solubility in liquids (according to Henry’s law) and from poor mass/heat transfer of reactants/products to/from the catalyst surface due to the physical separation of the phases and the low G-L– and L-S–specific interface areas (10^2^ to 10^3^ m^2^/m^3^) (Fig. 1, A and B). In practice, high temperatures and pressures are often required to promote the G-L-(S) contact that negatively affects the energy efficiency and safety of reactors. Efforts to date have focused on the design of advanced bubble generators (e.g., venturi, fluid oscillation, baffled agitation systems, and porous glass membranes) to increase the G-L–specific interface area and promote gas solubility in state-of-the-art packed bed (e.g., trickle beds) and slurry bubble column reactors (*3*, *4*). In addition, continuous flow G-L microreactors can generate very large surface areas, but they require complex equipment and are often difficult to upscale, especially in the presence of catalytic particles (Fig. 1C) (*5*, *6*). As a way out, microstructured G-L-(S) interfaces can be engineered to build G-L-(S) (catalytic) nanoreactors, allowing potential enhancement of reaction rates (Fig. 1, D to H, and Table 1). This perspective compiles possible strategies to engineer G-L-(S) interfaces and their credentials for the synthesis of commodity chemicals such as hydrogen peroxide, ammonia, and formic acid. These nanoreactors comprise (i) catalytic membrane contactors, (ii) microdroplets, (iii) micromarbles, (iv) microbubbles (including cavitation bubbles), and (v) particle-stabilized bubbles (microfoams).

**Table 1.** Taxonomy of microstructured G-L-(S) interfaces for chemical reactions. N/A, not applicable.

Object	Membrane contactors	Microdroplets	Particle-stabilized micromarbles	Microbubbles	Particle-stabilized microfoams
**Solvent**	Water	Water, organic solvents	Water, organic solvents/ionic liquid/liquid metal	Water, organic solvents	Water, organic solvents
**Stabilizer**	No	No	Hydrophobic (catalytic) particles	No	Hydrophobic (catalytic) particles
**Support**	Hydrophobic porous membrane	Hydrophobic material	Hydrophobic material	No	No
**Preparation**	Pouring the solvent on hydrophobic porous membrane/substrate	(Electro)spray methods at ambient conditions	Microdroplet spray on hydrophobic powder, droplet impact, droplet evaporation, electrostatic methods	Nozzle or ultrasound equipment	Magnetic/mechanical stirring or ultrasound using hydrophobic particles
**Size**	Nanometers to micrometers	<1000 μm	μm to cm	<100 μm	<1000 μm
**Lifetime**	N/A	Seconds/minutes	Hours	Seconds	Days/months
**Scale**	Grams to kilograms	Milligrams	Grams	Grams to kilograms	Grams to kilograms

## DISCUSSION

Membrane contactors (Fig. 1D) are typically made of hydrophobic porous polymer substrates [e.g., polytetrafluoroethylene (PTFE)–coated melamine foam, porous polyvinylidene difluoride, and polypropylene hollow fiber] or hydrophobic ceramic material and are designed to allow selective permeation of gas while preventing liquid from passing through. They can be implemented for both chemo- and electrocatalysis by incorporating catalytic centers. The substrates promote triphasic contact between the phases by generating microstructured interfaces within nanopores (e.g., thin liquid and gas films and confined interfaces). Membrane contactors have also been extensively used in gas-diffusing electrodes (GDEs) to enhance gas-electrode interaction. Hydrophobic substrates with rough surfaces can repel the electrolyte, creating thin gas films (and nanobubbles) between the electrode and the liquid electrolyte, commonly referred to as plastrons, based on Cassie-Baxter states. GDEs comprise a gas diffusion and a catalyst layer. The gas diffusion layer consists of a macroporous substrate and a microporous layer, often comprising a fluorinated polymer coating. Positioned between the microporous substrate and the catalyst layer, the microporous layer enhances interfacial electrical connectivity and prevents flooding within the GDE. Typically, the microporous layer is based on a blend of carbon black nanoparticles and a hydrophobic polymer. Microdroplets (Fig. 1E) are commonly generated using a nebulizer at high gas pressure in the size range of 1 to 100 μm and can be operated either as (electro)spray or on a hydrophobic substrate. Micromarbles (Fig. 1F) can be built by assembling surface-active (catalytic) particles (hydrophobic or oleophilic, 50 to 1000 nm) on microdroplets at the G-L interface, which markedly reduces liquid evaporation compared to bare microdroplets. Microbubbles (Fig. 1G) with a size ranging from 1 to 100 μm can be generated in water or organic solvents using flow nozzles and venturi devices. Last, microfoams (Fig. 1H) can be generated by assembling surface-active (catalytic) particles (hydrophobic or oleophilic, 50 to 400 nm) on microbubbles at the G-L interface.

Microstructured G-L-(S) interfaces introduce heterogeneous environments wherein molecules (including catalytic metal complexes and enzymes) can experience distinct interactions based on their location. The unique properties of microstructured G-L-(S) interfaces are depicted in Fig. 1 (I to L). Recent experiments and simulations demonstrate the tangible influence of high-surface electric fields (of the order of 10^9^ V/m) that can occur at microscale G-L interface due to preferential adsorption of HO^−^ species (*7*, *8*), making microdroplets behave as electrochemical “nanocells” (Fig. 1I) (*9*). These large interfacial electrical fields favor the formation of •OH radicals and carbocations (*10*, *11*). Moreover, preferential reagent and water orientation at the G-L (water) interface constitutes an additional means of enhancing the reactivity (Fig. 1J) (*12*, *13*). The interfacial acidity and basicity of water microdroplets can be much stronger than in the bulk phase, presumably due to limited hydration (*14*, *15*), which has implications on acid-base–catalyzed chemical reactions occurring at the air-water interface (Fig. 1K) (*14*). Last, when submerged in an aqueous medium, (super)hydrophobic particles and surfaces (e.g., PTFE) can ensnare thin gas films (and nanobubbles) between the substrate and the liquid, commonly referred to as plastrons, leading to an enhanced gas concentration within the G-L-S microenvironment (Fig. 1L) (*16*). The formation, stability, and dynamics of plastrons depend on the hydrophobic properties of the particles/surface, as well as on their micro/nanostructure and roughness.

Very recently, Wang and co-workers have reported contact-electro-catalysis (CEC) (Fig. 1M) that relies on an induced electron transfer between a solution and a hydrophobic (fluorinated) dielectric solid particle (e.g., PTFE) at the (G)-L-S interface using a mechanical stimulus such as ultrasound and ball milling (i.e., triboelectric effect) (*17*, *18*). Dielectric solid particles can promote electron transfer by the contact-electrification effect and input mechanical energy that can promote redox reactions. This concept has been demonstrated for the synthesis of H_2_O_2_ from H_2_O and O_2_ over PTFE (*19*–*21*). PTFE is capable of reducing O_2_ by the generation of reactive oxygen species (ROS) (i.e., hydroxyl and superoxide radicals) that can react by exchanging protons and electrons through the hydrogen bonds network of water, i.e., owing to the Grotthuss mechanism. ROS can be further used for the in situ oxidation of refractory organic compounds for wastewater treatment (*22*). CEC has also been designed combining TiO_2_ and CuBr_2_ using ultrasound as stimulus that can promote the atom transfer radical polymerization of methyl acrylate and ethyl α-bromoisobutyrate as initiator (*23*).

**Fig. 2.**
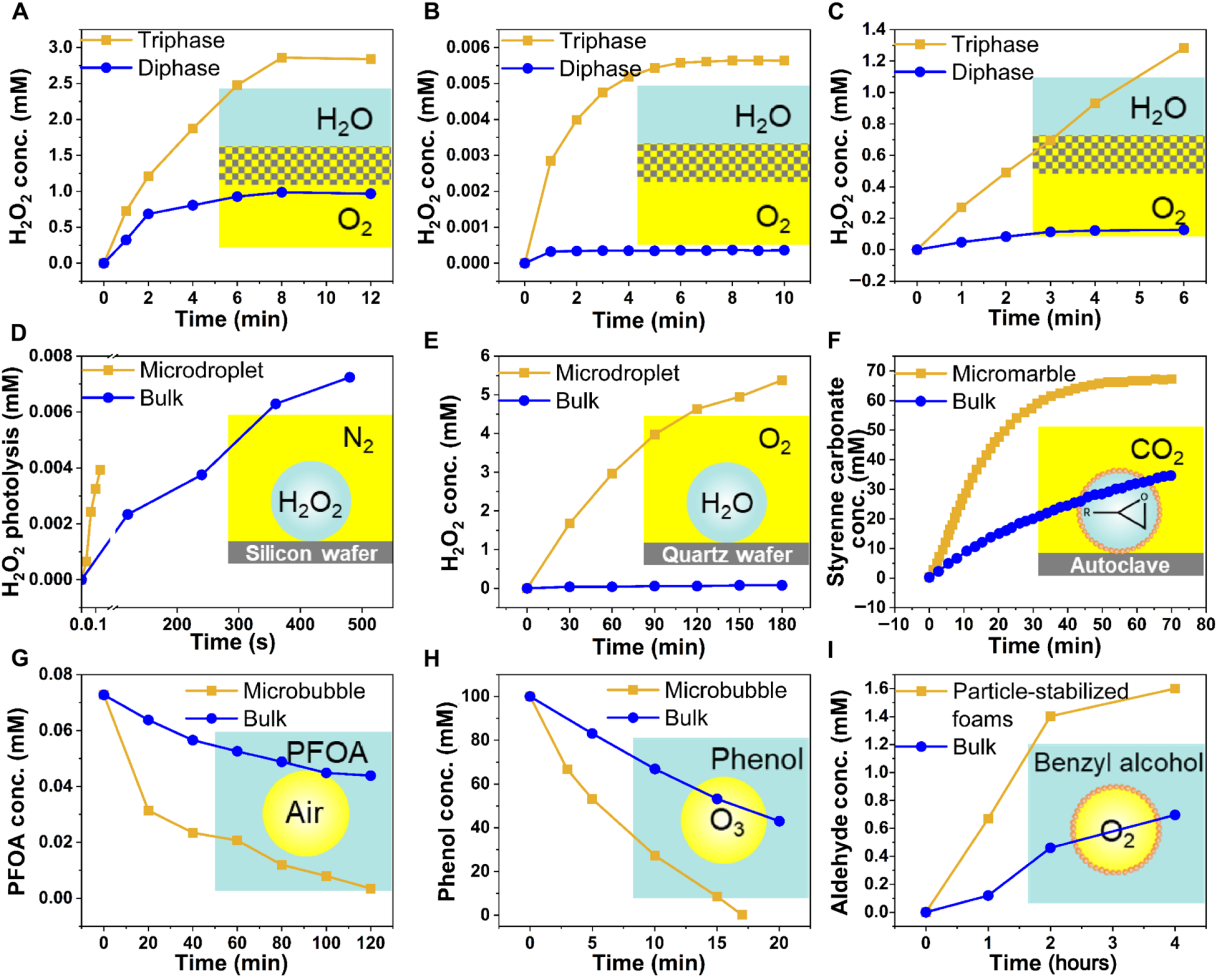
Representative kinetic plots for the synthesis of commodities in microstructured G-L-(S) interfaces. Entries in Table 2: (**A**) entry 1, (**B**) entry 2, (**C**) entry 3, (**D**) entry 7, (**E**) entry 10, (**F**) entry 13, (**G**) entry 15, (**H**) entry 17, and (**I**) entry 20.

Table 2 lists acceleration factors for microstructured G-L-(S) interfaces in membrane contactors, microdroplets, micromarbles, microbubbles, and particle-stabilized microfoams targeting the synthesis of commodity chemicals such as hydrogen peroxide (H_2_O_2_), ammonia (NH_3_), and formic acid (HCOOH), whereas Fig. 2 compiles representative kinetic plots. The most straightforward way to promote reactions at the G-L-(S) interface is by using a catalytic membrane contactor coupling a catalyst and a mesoporous membrane (ϕ = 5 to 20 nm) (*24*). By confining G-L interfaces in mesopores, membrane contactors can enhance the gas concentration in the vicinity of catalytic centers and thus accelerate reactions (*25*, *26*). Recently, three independent teams have reported the synthesis of H_2_O_2_ by reaction of air (O_2_) and water in porous hydrophobic substrates including an immobilized photocatalyst (e.g., carbon nitride), with acceleration factors between 3 and 11 compared to the reaction in bulk water (Table 2, entries 1 to 3) (*27*–*29*). The higher rates can be explained by a high air (O_2_) permeability combined with an enhanced air (O_2_) concentration near the photocatalytic particles/sheets promoted by the hydrophobic microenvironment that can enable the formation of plastrons. As a matter of fact, plastrons have been shown to enhance the activity and tune the selectivity of hybrid catalysts combining a hydrophobic support and a catalytic phase (e.g., PTFE-NiCo_2_O_4_) (*30*) or in hydrophobized electrocatalysts (e.g., hydrophobic zinc oxide or metal copper) (*31*). In both cases, hydrophobic moieties allow gas pre-concentration and enhanced surface diffusion of gas molecules/plastrons to the active centers. In electrochemistry, research on (superhydrophobic) GDEs holds significance. Electrosynthesis of H_2_O_2_ was achieved in a GDE consisting of carbon felt modified with PTFE acting as gas diffusion layer and substrate and carbon black loaded on the other side, by supplying air and the electrolyte on each side, with a rate of 0.907 mmol cm^−2^ hour^−1^ (Table 2, entry 4) (*32*). In contrast, only trace amounts of H_2_O_2_ were produced by fully immersing the electrode on the electrolyte. Electrosynthesis of NH_3_ was also demonstrated in a GDE based on a stainless-steel cloth promoting N_2_ and H_2_ diffusion and G-L contact in nonaqueous electrolytes. The reaction rate was 30 ± 5 nmol cm^−2^ s^−1^, whereas the highest reported rates obtained on copper foil electrodes are about 7.9 ± 1.6 nmol cm^−2^ s^−1^ (Table 2, entry 5) (*33*, *34*).

**Table 2.** Examples of reaction rate acceleration for different microstructured G-L-S interface systems. (i) Membrane contactors, (ii) microdroplets, (iii) micromarbles, (iv) microbubbles, and (v) particle-stabilized microfoams. Nomenclature: rt., room temperature; Mcells, one million cells; g_cat_, one gram catalyst; TEOA, Triethanolamine; HF, hydrogen fluoride.

Interface type	Entry	Reactions	Rate (bulk)	Rate (interface)	Acceleration factor
Membrane contactors	1	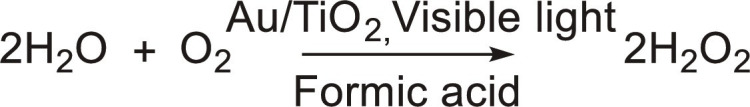	0.23 mM hour^−1^	0.7 mM hour^−1^	~3 (*27*)
	2	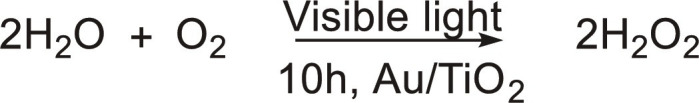	0.000567 mmol hour^−1^	0.00603 mmol hour^−1^	11 (*28*)
	3	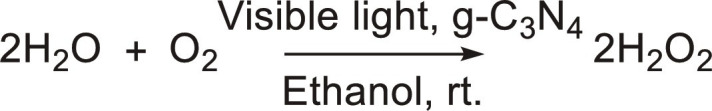	0.048 mM hour^−1^	0.375 mM hour^−1^	7.8 (*29*)
	4	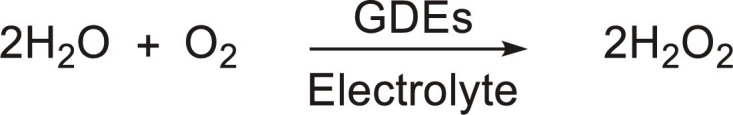	0.001 mmol cm^−2^ hour^−1^	0.907 mmol cm^−2^ hour^−1^	730 (*32*)
	5	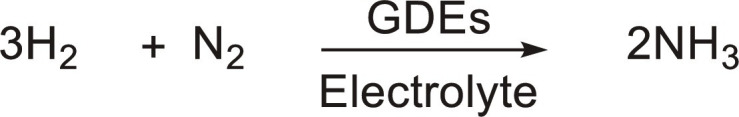	7.9 ± 1.6 nmol cm^−2^ s^−1^	30 ± 5 nmol cm^−2^ s^−1^	4 (*33*, *34*)
Microdroplets	6	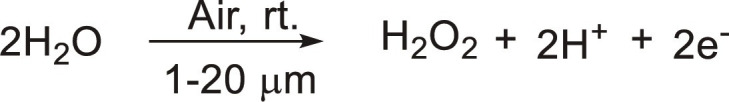	<detection limit	2500 mmol s^−1^	>57 (*11*)
			(4 mmol s^−1^)		
	7	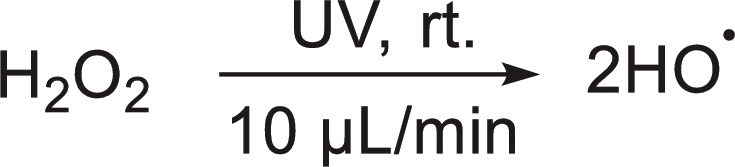	0.0001615 m s^−1^	0.31 m s^−1^	1900 (*41*)
	8	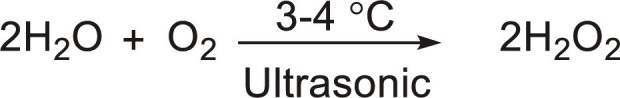	<detection limit	3.0 μM hour^−1^	>12 (*42*)
			(0.25 μM)		
	9	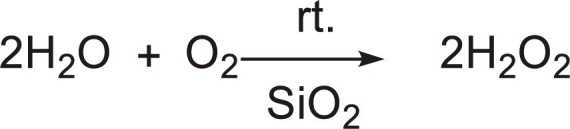	<detection limit	55.8 μM	220 (*44*)
			(0.25 μM)		
	10	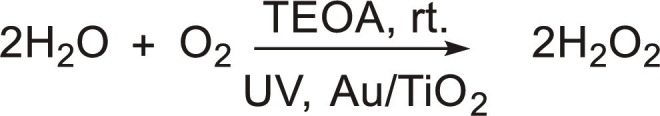	0.27 mmol *g*_cat_^−1^ hour^−1^	20.6 mmol *g*_cat_^−1^ hour^−1^	75 (*46*)
	11	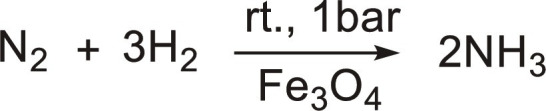	<0.068 nmol cm^−2^ s^−1^	32.9 ± 1.4 nmol cm^−2^ s^−1^	>480 (*47*)
	12	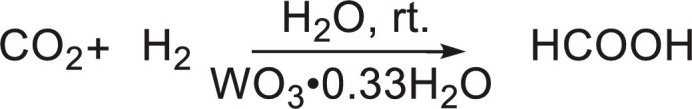	0.013 mmol hour^−1^ g^−1^	2.536 mmol hour^−1^ g^−1^	195 (*48*)
Micromarbles	13	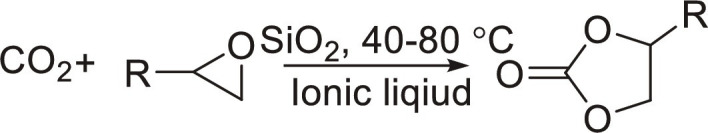	Conversion = 44%	Conversion = 95%	2 (*49*)
	14	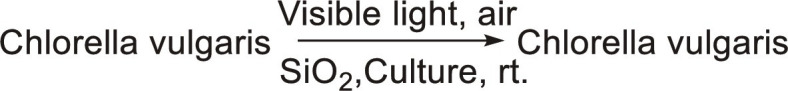	3.2 Mcells ml^−1^	93 Mcells ml^−1^	30 (*50*)
Microbubbles	15	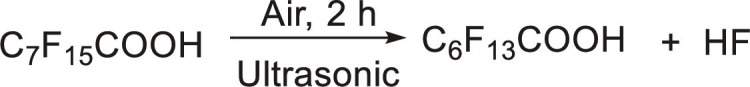	*k* = 0.26 hour^−1^	*k* = 0.90 hour^−1^	3.3 (*52*)
	16	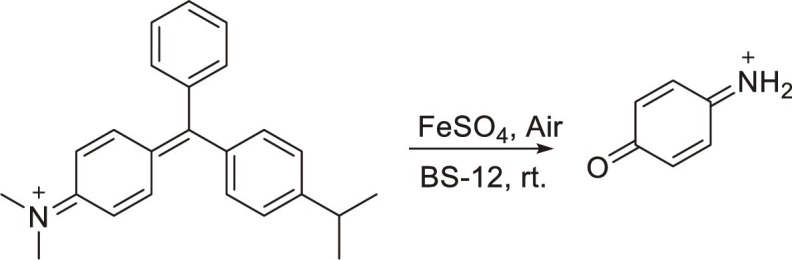	N/A	N/A	2.4 (*53*)
	17	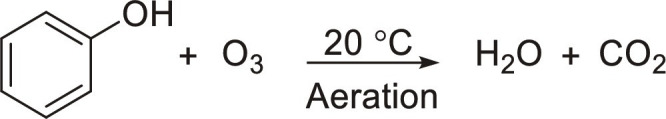	2.2 hour^−1^	10 hour^−1^	4.3 (*54*)
	18	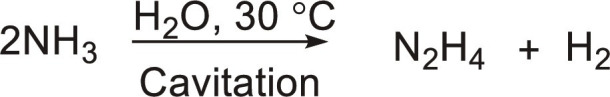	<detection limit	0.12 mM hour^−1^	N/A (*56*)
Microfoams	19	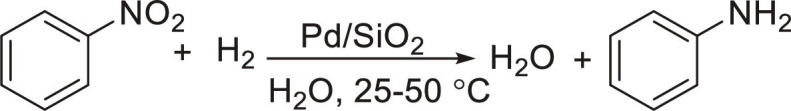	282 mol mol^−1^ hour^−1^	1440 mol mol^−1^ hour^−1^	5.1 (*57*)
	20	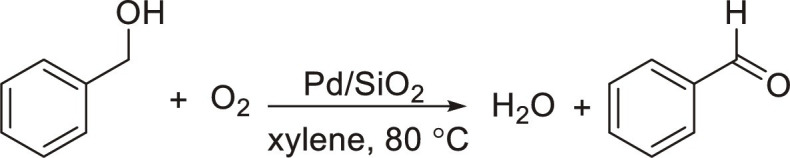	794 mol mol^−1^ hour^−1^	4502 mol mol^−1^ hour^−1^	5.7 (*58*)

Water microdroplets (<10 μm) can also accelerate the kinetics of reactions on G-L-(S) interfaces compared to the bulk phase, with particular relevance in photo/biological chemistry, environmental catalysis, and chemical synthesis (*35*–*38*). Notably, key synthetic reactions encompassing addition, condensation, elimination, substitution, redox, rearrangement, and non-covalent complexation experience acceleration when the reagents, especially polar molecules, reside at the G-L interface in water microdroplets (*39*). Recently, Zare and co-workers (*11*, *40*) demonstrated that H_2_O_2_ can be produced on aerosol water microdroplets without catalyst in either pure N_2_, air, O_2_, or ozone with acceleration factors up to 57 compared reactions in bulk water driven by the formation of •OH radicals at the microstructured G-L interface (Table 2, entry 6). Chu and co-workers (*41*) demonstrated that H_2_O_2_ photolysis at the air-water interface of microdroplets is 1900 times faster than that in bulk water (Table 2, entry 7). Mishra and co-workers (*42*) reported that different spray types can affect the rate of H_2_O_2_ generation, with ultrasonics playing a pivotal role rather than evaporation, to drive H_2_O_2_ production most likely due to the generation of cavitation microbubbles promoted by ultrasonication (Table 2, entry 8). Sliding water microdroplets on a PTFE surface can induce CEC based on triboelectric nanogeneration and promote photocatalytic production of ROS (e.g., •OH and •O_2_^−^ radicals) under ultraviolet (UV) and O_2_ without semiconductor (*43*). Very recently, Zare and co-workers (*44*) also demonstrated that CEC can occur between immobilized water microdroplets and that a hydrophilic dielectric solid surface can lead to H_2_O_2_ generation without need of mechanical stimulus such as ultrasound or droplet sliding (Table 2, entry 9). Using silica as a model solid, H_2_O_2_ generation was confirmed by mass spectrometric detection of ^18^O-labeled silanol groups (SiOH). The results pointed out that H_2_O_2_ was generated by •OH radical recombination without the need of dissolved O_2_, encompassing partial charge of the solid together with water acidification. In addition, Zare and co-workers (*45*) exemplified the conversion of benzoic acid into phenol using ^18^O-labeled H_2_O microdroplets, revealing that the hydroxyl group in phenol originates from hydroxyl radicals generated at the microdroplet-air interface.

Microdroplets can also interact with solid catalysts (not dielectric) to accelerate reactions. Three examples have been reported: (i) H_2_O_2_ photosynthesis under UV light combining Au/TiO_2_ and 300-μm microdroplets showing two times increase of the H_2_O_2_ evolution rate compared to the reaction in bulk phase (Table 2, entry 10) (*46*); (ii) NH_3_ synthesis at room temperature and atmospheric pressure with neither an external electric potential nor irradiation, where water microdroplets act as hydrogen source for N_2_ reduction in contact with Fe_3_O_4_, resulting in an acceleration factor higher than 480 compared to the reaction in bulk phase (Table 2, entry 11) (*47*); and (iii) photocatalytic CO_2_ reduction to HCOOH over WO_3_·0.33H_2_O using water microdroplets with an acceleration factor of 195 compared to the reaction in bulk phase, in the absence of sacrificial agents (Table 2, entry 12) (*48*). Solid catalysts can also be self-assembled at the G-L interface generating armored microdroplets (i.e., liquid micromarbles). Marbles can be generated with catalytically active or stimuli-responsive particles, which can promote the reaction rate. Zhang and co-workers (*49*) developed ionic liquid micromarbles stabilized by silica particles modified with dichlorodimethylsilane for the catalytic cycloaddition of CO_2_, and the reaction rate was doubled (Table 2, entry 13). In addition, Nguyen *et al.* (*50*) prepared microalgal culture liquid marbles stabilized by 10- to 20-nm silica particles as a photobioreactor which provides a 30-fold increase in maximum cell density as compared with the culture flask platform (Table 2, entry 14).

Microbubbles offer an alternative approach to design reactions occurring on G-L-(S) interfaces. Microbubbles have been less studied than microdroplets due to their more complex stabilization and handling ascribed to their lower density than liquids and buoyancy effects. Air microbubbles can develop notable interfacial charge by underwater contraction and induce the generation of •OH radicals upon collapse (*51*), making them suitable for applications in chemical reactions. For instance, perfluorooctanoic acid can be decomposed 3.3 times faster in a needle-plate pulsed discharge reactor incorporating microbubbles than in control oxidation experiments without microbubbles (Table 2, entry 15) (*52*). Recalcitrant organic pollutants in water can be oxidized by the Fenton reaction using microbubbles with an acceleration factor of 2.4 when compared with the reaction without microbubbles (Table 2, entry 16) (*53*). Yu and co-workers (*54*) conducted the decomposition of pollutants (e.g., phenol) by ozonation using air microbubbles (<50 μm) and conventional bubble aeration with macrobubbles (>1 mm). Ozonation with microbubbles was much more efficient with an acceleration factor of ~4.3 compared to conventional bubble aeration (Table 2, entry 17). The authors argued about a possible higher ozone concentration in the liquid film in microbubbles relying on •OH radical scavenger experiments. Opposing this view, Brookes and co-workers (*55*) reported no substantial evidence on a higher •OH radical production induced by microbubbles in water compared to standard aeration systems with ozone. They proposed that •OH generation is primarily connected to ozone self-decomposition, suggesting that alternative mechanisms for •OH production, such as microbubble collapse, may be of negligible significance or absent under the pH conditions examined and within the prevailing bubble size distribution. In addition to air or ozone microbubbles, Jérôme and co-workers (*56*) used cavitation bubbles generated by ultrasound irradiation on aqueous NH_3_ at a high frequency (525 kHz, 0.17 W/ml) to convert NH_3_ into hydrazine (Table 2, entry 18). The cavitation microbubbles served as nanoreactors, activating and transforming NH_3_ into NH species without the need of a catalyst. This method yielded hydrazine at the G-L interface preventing its decomposition.

Particle-stabilized microbubbles and microfoams can be generated using surface-active (catalytic) particles, offering the advantages of lower energy requirements and higher environmental sustainability. Surface-active particles can be easily recovered and separated from the reaction products, facilitating recycling and minimizing waste. Yang *et al.* showed that particle-stabilized aqueous foam can be used for nitrobenzene hydrogenation with around two to five times acceleration factors compared to the reaction in bulk water (Table 2, entry 19) (*57*). Recently, particle-stabilized oil foams have been designed with 5 to 10 times higher reaction rates in the aerobic oxidation of aromatic and aliphatic alcohols at low particle concentration (1 to 2 wt %) (Table 2, entry 20) (*58*). However, the benefits of particle-stabilized bubbles and microfoams to prepare commodity chemicals, driven most likely by plastrons, need yet to be demonstrated and constitutes a field with high potentials and foreseen future developments.

The examples above illustrate the potentials of microstructured G-L-(S) interfaces to enhance the rate of reactions compared to conventional bulk (catalytic) systems. Among the different concepts, membrane reactors encounter the drawbacks of high cost, scale-up challenges, hydrophobic coating fouling or degradation over time, and mechanical fragility. Specifically, fouling constitutes the primary challenge of membrane contactors, which can lead to decreased efficiency and increased operational costs due to the need for frequent cleaning or membrane replacement. However, membrane contactors can allow the formation of plastrons that accelerate the rate of reactions and be used as supports for conceiving CEC applications using fluorinated polymers or polymer-grafted inorganic supports using ultrasound as stimulus. Microdroplets can exhibit high surface area-to-volume ratios and excellent acceleration factors, but achieving large-scale production of microdroplets is not straightforward. It requires expensive equipment for their manufacture and size control, and microbubbles have a short lifetime due to their weak metastable nature. Microbubbles have limited application because intense sonication can induce cavitation, which may cause the breaking down of organic compounds and result in poor selectivity. Micromarbles can, to some extent, prolong the lifetime of microdroplets but show challenges associated to their size control and the complexity of the manufacture process, and the fabrication and manipulation of liquid micromarbles can be challenging. In this view, scaling up the manufacture of microdroplets, microbubbles, and micromarbles from the laboratory to real application remains a formidable challenge. In contrast, particle-stabilized microfoams with higher stability, reaching, in some cases, several months, can provide a wide range of potential applications. Furthermore, the amount of reagents required in particle-stabilized microfoams can be adjusted as desired, offering flexibility and control over reaction conditions. Microfoams increase markedly the contact area between the liquid and gas phases, which further enhances reaction efficiency. An additional advantage of microfoams is their compatibility with both aqueous and organic solvents, allowing easy implementation in state-of-the-art multiphase reactors, expanding accordingly the range of potential applications. Crucially, microfoams can be stabilized with a broad variety of surface-active particles that can enhance the gas concentration near catalytic centers driven by plastrons and promote CEC phenomena that need yet to be explored.

As key development, plastrons generated by entrapped gas on hydrophobic, rough particles while submerged in a liquid can serve as a means of gas transportation within a bulk liquid, allowing to overcome Henry’s law for gas solubility in liquids (*59*). This property, which is analogous to that observed in porous liquids, can enhance the activity for O_2_ electroreduction (*60*). We envision that plastrons on hydrophobic particles can also be implemented to other reactions such as CO_2_ electroreduction to hydrocarbons or methanol and the electrochemical synthesis of NH_3_ from N_2_ and H_2_ by carefully engineering microstructured G-L-(S) interfaces (*61*).

Dielectric particles can assemble at the G-L interface and generate microfoams under sonication or mechanical stirring, which provides an appropriate condition for CEC. However, microfoam systems present challenges, such as the need to design particles with suitable size, hydrophobicity, surface roughness, catalytic centers, and the convenience of recycling. Fine control of particle design can allow the location and orientation of catalytic centers at the G-L interface, thus enhancing the local G-L miscibility and, in turn, tuning the activity and selectivity of reactions. As a key advantage, microfoams do not require the use of pre-formed membranes as in the case of membrane contactors, reducing the cost. Besides, unlike microdroplet and micromarble systems, microfoams can be generated without intricate workup and high energy use. Microfoams can be stabilized through straightforward mechanical stirring, which also incurs lower energy utilization compared to ultrasonication methods used in microbubble systems that often suffer from low energy efficiency, especially in bubble cavitation systems (*62*).

While each type of microstructured G-L-(S) nanoreactor has its own set of strengths and weaknesses, we anticipate that particle-stabilized microfoams are the most promising option for future industrial applications due to their superior compatibility with different nanoscopic phenomena. They can seamlessly integrate contact-electrification effects, plastrons, and even microporous water. Another avenue for technological advancement involves the design of continuous or semicontinuous reactors implemented with particle-stabilized microfoams. These systems require a dedicated optimization of particle size, hydrophobicity, surface roughness, and catalytic centers for each specific reaction.

## OUTLOOK

In summary, the recent encouraging findings on microstructured G-L-(S) nanoreactors highlighted in this perspective open an avenue toward the reengineering of multiphase reactors to access commodity chemicals with superior reaction rates and efficiency. Additional applications of microstructured G-L-(S) interfaces for chemical transformations in pharmaceuticals, the manufacture of bulk chemicals, and organic synthesis are also foreseeable and encouraging. Among the possible strategies to shape G-L-(S) interfaces, particle-stabilized (catalytic) microfoams emerge as the most promising G-L-(S) nanoreactors with high versatility and easy implementation to reengineer state-of-the-art multiphase reactors. Besides, plastrons entrapped in particles reveal as a useful means to transport gases within liquids to enhance the rate in electrochemical reactions for the synthesis of commodities.
